# Association of Behavioral and Socioeconomic Factors With Sexually Transmitted Infection Positivity in Vulnerable Women From a Neotropical Setting

**DOI:** 10.1155/ipid/4600610

**Published:** 2025-11-20

**Authors:** Gabriel Rodrigues Côra, José de Ribamar Ross, Flávia Castello Branco Vidal, Iagho José Lima Diniz, Maria Edileuza Soares Moura

**Affiliations:** ^1^Graduate Program in Biodiversity, Environment and Health, State University of Maranhão, Caxias, Brazil; ^2^Department of Health Sciences, State University of Maranhão, Caxias, Brazil; ^3^Department of Morphology, Federal University of Maranhão, São Luís, Brazil; ^4^Graduate Program in Bioinformatics, Federal University of Paraná, Curitiba, Brazil

**Keywords:** quilombola, Roma, risk factors, rural population, sexually transmitted infections, vulnerable populations

## Abstract

**Background:**

Sexually transmitted infections (STIs) represent a global public health concern, particularly among vulnerable populations. In Brazil, women from quilombola, Roma, and rural communities face increased risks due to socioeconomic and cultural conditions that exacerbate susceptibility to these infections. Furthermore, they are frequently underrepresented in scientific studies, leading to a limited understanding of the epidemiological characteristics of STIs within these groups. This study aimed to estimate the prevalence of STIs among women in vulnerable situations in a neotropical region of Brazil and to analyze the association between the pathogen presence, symptoms, behavioral risk factors, and socioeconomic aspects.

**Methods:**

A cross-sectional study was conducted in eastern Maranhão, in the municipality of Caxias, involving 295 quilombola, Roma, and rural women. Data were collected using a structured questionnaire addressing sociodemographic and behavioral factors, along with cervical liquid-based cytology samples. The samples were tested using real-time PCR with the Seegene Allplex STI Essential Assay kit. Statistical analysis employed both descriptive and inferential methods, including chi-square and Fisher's exact tests, as well as unconditional univariate and multivariate logistic regression models.

**Results:**

The overall prevalence of STIs was 72.9% (95% CI: 66.8–77.3), with *Ureaplasma parvum* being the most common pathogen (61.7%), followed by *Mycoplasma hominis* (35.3%). *Trichomonas vaginalis* was significantly associated with vaginal pruritus (OR = 2.566, *p*=0.047). Behavioral and socioeconomic factors associated with the presence of STIs included the absence of previous cytology screening (AOR = 2.39, *p*=0.046), high frequency of daily bathing (AOR = 3.28, *p*=0.042), and prolonged use of tight underwear (AOR = 1.83, *p*=0.027).

**Conclusion:**

This study revealed a high prevalence of STIs among quilombola, rural, and Roma women. The findings highlight the complexity of sexual and reproductive health in these communities and underscore the need for tailored, culturally sensitive approaches in public health policies.

## 1. Introduction

Sexually transmitted infections (STIs) represent a major global public health issue, with significant prevalence and impact on individuals' quality of life, affecting both sexes and increasing vulnerability to other diseases. The magnitude of STIs, coupled with difficulties in accessing timely diagnosis and appropriate treatment, as well as the underreporting of cases, perpetuates the chain of transmission. The diversity of etiological agents and the multiple associated clinical syndromes further complicate the diagnosis and management of STIs [1, 2].

STIs are caused by a wide range of pathogens, including bacteria, viruses, fungi, and protozoa. Among the most common are human papillomavirus (HPV), *Chlamydia trachomatis*, *Neisseria gonorrhoeae*, herpes simplex virus Type 2, *Mycoplasma hominis*, *M. genitalium*, *Ureaplasma urealyticum*, *U. parvum*, and *Trichomonas vaginalis*. Transmission occurs primarily through sexual contact, including vaginal, anal, and oral intercourse, particularly in the absence of condom use. In addition, some STIs can also be transmitted through blood or blood products and from mother to child during pregnancy and childbirth, as in the case of chlamydia, gonorrhea, Hepatitis B, HIV, and syphilis [3–5].

From an epidemiological perspective, the magnitude of this global health problem is evident. According to the World Health Organization (WHO), more than one million STIs are acquired every day. In 2020, the WHO estimated 374 million new infections with one of four major STIs: chlamydia (129 million), gonorrhea (82 million), syphilis (7.1 million), and trichomoniasis (156 million). It is estimated that 300 million women are infected with HPV, the leading cause of cervical cancer [6]. In the Americas, an estimated 74 million STI cases were reported. In Brazil, the actual epidemiological situation of STIs and their complications are underestimated due to the lack of mandatory reporting for most infections and the scarcity of sentinel and population-based studies [1]. A study conducted by the Ministry of Health in 2008 assessing STI prevalence in specific populations across six Brazilian capitals revealed high rates, with 14.4% of STIs being bacterial and 41.9% viral [7].

Several factors influence the emergence, dissemination, and persistence of STIs. These may be biological, psychological, behavioral, or social in nature and include early sexual debut, low condom use, inadequate genital–anal hygiene, new or multiple sexual partners, contraceptive use, tight clothing, alcohol or drug consumption, hormonal alterations, immunodeficiency, use of antibiotics or vaginal medication, urinary incontinence, stress, and socioeconomic or health-related vulnerabilities [8, 9]. It is undeniable that these infections do not affect the population equally as they disproportionately impact vulnerable groups.

In Brazil, women from quilombola, Roma, and rural communities face vulnerabilities related to living conditions, individual and collective factors, and social, economic, and cultural aspects that expose them to specific health risks and increase their susceptibility to acquiring STIs. Although ethnic and racial disparities in relation to STIs are frequently studied, research addressing this issue among these minoritized groups of women in Brazil remains scarce [10–13].

Rural, quilombola, and Roma communities are often underrepresented in scientific studies, resulting in a limited understanding of the epidemiological characteristics of STIs within these groups. Previous studies have demonstrated the presence of STIs in quilombola, rural, and Roma communities, with prevalence rates higher than those observed in the general population [10, 14–18]. This is the first study to estimate the prevalence of STIs among women in vulnerable situations in a neotropical region of Brazil, analyzing the associations between pathogen presence, symptoms, behavioral risk factors, and socioeconomic aspects.

## 2. Methods

### 2.1. Study Design and Setting

This study utilized a cross-sectional, exploratory design with a quantitative approach. It was conducted in eastern Maranhão, specifically in the municipality of Caxias, which has a Municipal Human Development Index of 0.624 [19]. The research included women from six quilombola communities: Jenipapo, Planaçu, Lavras, Mimoso, Soledade, and Lagoa dos Pretos/Centro da Lagoa. Additionally, a Roma community located in a peripheral area known as Pé do Morro, within the Vila Arias neighborhood, and a rural community named the village of Caxirimbu were also included.

### 2.2. Population and Sample

A total of 1256 women aged 18–64 years were identified through the records of community health agents within these communities. This population served as the foundation for calculating and defining the study sample size. With a 95% confidence interval (95% CI) and a 5% margin of error, the final sample comprised 295 women.

### 2.3. Inclusion and Exclusion Criteria

The inclusion criteria for the study were as follows: women who had engaged in vaginal intercourse; women aged between 18 and 64 years, 11 months, and 29 days; residents of the communities where the study was conducted; women belonging to families from quilombola areas certified by INCRA and/or the Palmares Cultural Foundation; women from Roma families; and women from rural families.

The exclusion criteria were as follows: women with any mental or cognitive impairments that prevented completion of the research questionnaire; a history of genital surgeries such as total hysterectomy; menstruation at the time of or within 1 week prior to sample collection; pregnancy with threatened abortion or premature rupture of membranes; use of medications that could affect the vaginal microbiota, such as antibiotics or antifungals, or use of intimate hygiene products within 48 h prior to sample collection; sexual activity within 48 h prior to collection; and cervical–vaginal smears considered unsatisfactory or samples showing DNA degradation.

### 2.4. Data Collection and Gynecological Examination

The study activities were conducted on dates previously arranged with community health agents and local health teams, in accordance with established plans and schedules, and with the support of community leaders. The community health agents were responsible for inviting eligible women. Within each community, participant selection was considered random, as any woman meeting the eligibility criteria and recommendations could be selected.

Upon consent, participants signed the informed consent form and subsequently completed a structured questionnaire containing 30 questions, divided into three sections: sociodemographic characteristics, sexual behavior and practices, and hygiene habits. The questionnaire was administered through individual interviews.

Following the interviews, participants underwent cervical cytology collection in private rooms, either at the reference health unit in their area or in adapted spaces within the communities, such as classrooms provided by schools. The furniture and equipment for cytology collection were supplied by the Primary Health Care Coordination of Caxias (MA), including cytopathology request forms, cytology kits, examination tables, procedural gloves, appropriate fixatives, disposable surgical masks, examination and patient drapes, and gauze.

For cervical cytology, secretions from the ectocervix and endocervix were collected using the liquid-based cytology method, following the manufacturer's instructions and standardized collection protocols of the Brazilian Ministry of Health [20]. Cervical samples were then transferred to the Molecular Biology Laboratory of the Federal University of Maranhão for the identification of STI etiological agents using the polymerase chain reaction (PCR).

### 2.5. Molecular Detection of STI Agents

For PCR preparation, samples were transferred to 1.5-mL microtubes and centrifuged at 13,000 rpm for 15 min (Kasvi Mini Centrifuge K14-1215). The supernatant was discarded, leaving the pellet at the bottom of the tube. Subsequently, 500 μL of Seegene Universal Lysis Buffer (LB) was added to carefully resuspend the pellet using a pipette, avoiding foam formation.

Genetic material extraction was fully automated using the Seegene Hamilton Nimbus IVD system and the Seegene STARMag 96 X 4 Universal Cartridge Kit, which includes four steps: sample lysis, nucleic acid binding to magnetic beads, debris washing, and elution of purified nucleic acids. Real-time PCR (qPCR) was performed on the extracted and purified genetic material using the Bio-Rad CFX96 IVD platform.

For STI analysis, the Seegene Allplex STI Essential Assay kit was employed, which is an in vitro qualitative test that detects *Chlamydia trachomatis* (CT), *N. gonorrhoeae* (NG), *M. genitalium* (MG), *M. hominis* (MH), *U. urealyticum* (UU), *U. parvum* (UP), and *T. vaginalis* (TV) from both genital swabs and liquid-based cytology specimens. The kit components include 4X STI-EA MOM (amplification and detection reagents: primers and probes); EM1 (containing DNA polymerase, uracil-DNA glycosylase [UDG], and buffer with dNTPs); STI-EA PC (positive control with a mixture of pathogen clones); IC (internal control); and RNase-free water.

For PCR mix preparation, 5 μL of 4X STI-EA MOM, 5 μL of EM1, 5 μL of RNase-free water, and 5 μL of purified nucleic acid were combined, totaling 20 μL per reaction. The mixture was vortexed and briefly centrifuged. Aliquots of 15 μL of PCR Master Mix were dispensed into PCR tubes, followed by the addition of 5 μL of nucleic acid from each sample. For the negative control (NC), 5 μL of RNase-free water was used instead of nucleic acid. For the positive control (PC), 5 μL of STI-EA PC was added.

The amplification step was performed in cycles, each consisting of denaturation, annealing, and extension phases, following the temperatures and times specified for the reaction. The qPCR assay was configured as follows: initially, the reaction was maintained at 50°C for 4 min, followed by heating to 95°C for 15 min. Subsequently, five cycles were performed at 95°C for 30 s, 60°C for 1 min, and 72°C for 30 s. This was followed by 40 cycles at 95°C for 10 s, 60°C for 1 min, and 72°C for 10 s. Plate reading was configured for steps 7 and 8. Fluorescence was detected at 60°C and 72°C. qPCR reaction data were analyzed using the Seegene Viewer software.

### 2.6. Statistical Analysis

Statistical analysis began with descriptive analyses, generating frequency tables of sociodemographic and behavioral variables, as well as the prevalence of microorganisms identified by PCR, highlighting absolute and relative frequencies with their respective 95% CIs. Subsequently, simple logistic regression was performed to assess the association between vaginal symptoms and STI pathogen positivity.

To investigate the influence of behavioral factors and socioeconomic aspects on STI positivity, association analyses were initially conducted using chi-square or Fisher's exact test to determine potential independent variables associated with the dependent variable. Variables were evaluated according to their *p* values, odds ratios (ORs), and 95% CIs, with selection guided by the literature review and theoretical relevance.

For the construction of the unconditional multivariate logistic regression model, variables were first selected based on their statistical significance in the bivariate analyses. Variables with *p* ≤ 0.20 were included in the multivariate model. The model was adjusted to evaluate the combined impact of these variables. The final analysis was refined by retaining only those variables with *p* ≤ 0.05 to optimize robustness. Statistical analyses were performed using SPSS software, Version 21.0.0.

### 2.7. Ethical Considerations

This study was conducted in accordance with the Declaration of Helsinki (1964) and complied with the guidelines established in Resolution No. 466/12 of the Brazilian National Health Council for research involving human subjects. The study protocol was approved by the Research Ethics Committee of the Center for Higher Studies of Caxias (Approval Number 6.335.876).

## 3. Results

A total of 295 women participated in this study, comprising 129 (43.7%) quilombolas, 154 (52.2%) rural women, and 12 (4.1%) Roma. Regarding sociodemographic characteristics, the participants had a mean age of 37 years (SD: 13.15; 95% CI: 35.65–38.43), with the 30–44 age group being the most frequent (111/37.6%). The majority self-identified as mixed race (149/50.5%), were married or in a stable union (196/66.4%), had completed up to primary education (81/27.5%), were Catholic (222/75.3%), and reported agriculture as their primary occupation (236/80.0%) for subsistence. Additionally, most participants reported a family income below the minimum wage (179/60.7%) (Table 1).

Regarding the risk factors, most participants reported not consuming alcohol (190/64.4%) and not being smokers (264/89.5%). We also observed that the majority had their first sexual intercourse (coitarche) before the age of 18 (221/74.9%). The mean age at sexual debut was 16 years (SD: 2.69; 95% CI: 15.68–16.33). A higher proportion of women reported having had more than one sexual partner in their lifetime (185/62.7%), not using contraceptives (243/82.4%), and not using condoms (192/65.1%). In addition, most women reported no previous history of STIs (275/93.2%) (Table 2).

Regarding the symptomatology, 52.2% (*n* = 154) of the participants reported vaginal discharge and pelvic pain. Vaginal pruritus was mentioned by 20.7% (*n* = 61). Dysuria was reported by 13.9% (*n* = 41), and 22.0% (*n* = 65) of the women reported dyspareunia (Table 2).

In terms of hygiene practices, most women reported bathing three to four  times per day (215/72.9%) and performing intimate hygiene during every bath (250/84.7%). The majority stated they used regular soap (156/52.9%) and changed their underwear more than once a day (278/94.2%). When asked about vaginal douching, most participants reported not practicing it (226/76.6%) (Table 2).

**Figure 1:**
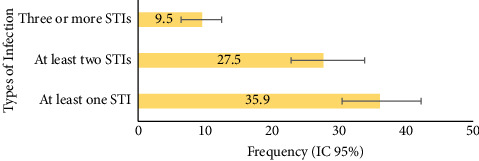
Percentage distribution of STIs by the type of infection.

In the molecular analysis, samples from 295 women were examined to determine the prevalence of infections caused by various STI pathogens. The results showed that 72.9% (*n* = 215; 95% CI: 66.8–77.3) tested positive for at least one etiological agent. Overall, 106 women (35.9%; 95% CI: 30.3–42.0) were diagnosed with at least one confirmed STI. In addition, 81 participants (27.5%; 95% CI: 22.7–33.6) were diagnosed with two different STIs, while 28 women (9.5%; 95% CI: 6.4–12.4) presented with three or more STIs (Figure 1).

The overall prevalence of STIs was higher among rural women (53.0%; *n* = 114), followed by quilombola women (43.3%; *n* = 93) and Roma women (3.7%; *n* = 8). Regarding the type of infection, 51.9% (*n* = 55) of cases with at least one STI occurred among rural women, compared to 44.3% (*n* = 47) among quilombola women and 3.8% (*n* = 4) among Roma women. For two concurrent STIs, 56.8% (*n* = 46) of cases were observed among rural women, 40.7% (*n* = 33) among quilombola women, and 2.5% (*n* = 2) among Roma women. For cases involving three or more STIs, the prevalence was 46.4% (*n* = 13) among both rural and quilombola women and 7.1% (*n* = 2) among Roma women.

**Figure 2:**
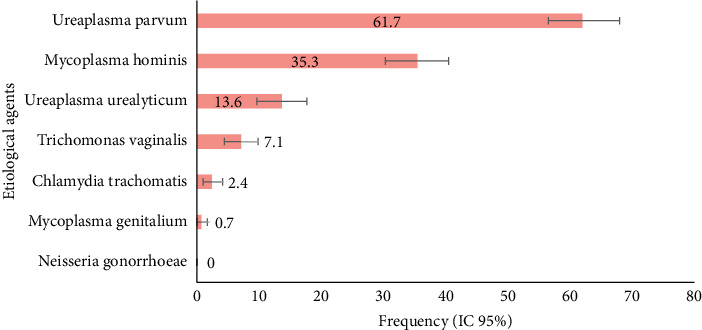
Prevalence of etiological agents of STIs among quilombola, Romani, and rural women in 295 samples examined.

The most prevalent etiological agent was *U. parvum*, detected in 182 participants (61.7%; 95% CI: 56.3–67.7), followed by M. hominis, present in 104 cervical samples (35.3%; 95% CI: 30.2–40.3). Other etiological agents, such as *U. urealyticum, T. vaginalis, C. trachomatis, and M. genitalium* were less frequent, occurring in 40 (13.6%; 95% CI: 9.6–17.6), 21 (7.1%; 95% CI: 4.4–9.8), 7 (2.4%; 95% CI: 1.0–4.1), and 2 (0.7%; 95% CI: 0–1.7) women, respectively. No cases of *N. gonorrhoeae* infection were found among the participants (Figure 2).

The distribution of STI etiological agents varied across the different groups. Among the cases *of C. trachomatis*, 85.7% (*n* = 6) occurred in rural women, while 14.3% (*n* = 1) were found in quilombola women, with no cases detected among Roma women. All cases of *M. genitalium* (100%; *n* = 2) were found among quilombola women, while *M. hominis* was distributed among rural (50.0%; *n* = 52), quilombola (45.2%; *n* = 47), and Roma women (4.8%; *n* = 5). For *T. vaginalis*, 52.4% (*n* = 11) of detections occurred in rural women and 47.6% (*n* = 10) in quilombola women, with no cases in Roma women. Infections with U. parvum were predominant among rural women (56.0%; *n* = 102), followed by quilombola (40.7%; *n* = 74) and Roma women (3.3%; *n* = 6). *U. urealyticum* was more frequently detected in quilombola women (52.5%; *n* = 21), followed by rural (40.0%; *n* = 16) and Roma women (7.5%; *n* = 3).

In the bivariate analysis of the association between different vaginal symptoms and the positivity for etiological agents of STIs, only vaginal pruritus and T. vaginalis showed a significant association. The positivity rate was 13.1% (*n* = 8), with OR = 2.566 and *p* value = 0.047. This indicates that women with *T. vaginalis* are 2.566 times more likely to present vaginal pruritus. The remaining results did not demonstrate significant associations, indicating that there is insufficient evidence to link the studied symptoms with the etiological agents of STIs among the participating women (Table 3).

In the bivariate analysis, the variables potentially associated with genital infection were identified based on a *p* value threshold of 0.20. This threshold was chosen as it allows for a broader exploration of potential associations, which is particularly important in the early stages of research. The variables that met this criterion were previous cytology screening (*p*=0.033), number of daily baths (*p*=0.114), and wearing underwear during the day (*p*=0.026). Other variables, such as age group, occupation, educational level, marital status, household income, alcohol consumption, smoking, age at first sexual intercourse, previous history of STIs, vaginal symptoms, number of sexual partners, condom use, contraceptive use, intimate hygiene, products used for intimate hygiene, and sitz baths, did not meet this threshold and therefore were not considered significant in this analysis (Table 4).

After adjustment in the multiple logistic regression model, the independent variables significantly associated with at least one STI were prior cytology screening (OR = 2.39, 95% CI: 1.01–5.62, *p*=0.046), number of daily baths (OR = 3.28, 95% CI: 1.04–10.33, *p*=0.042), and wearing underwear for most of the day (OR = 1.83, 95% CI: 1.07–3.12, *p*=0.027). These results carry significant implications. They indicate that women who had never undergone cytology screening were 2.39 times more likely to have an STI. Likewise, women who reported taking five or more baths per day had 3.28 times higher odds of having an STI compared with those who reported taking one to two baths per day. In addition, women who wore underwear for most of the day presented an increased risk of STIs. These findings underscore the importance of regular cytology screening and the potential impact of daily hygiene practices on STI risk (Table 4).

## 4. Discussion

The Global Health Sector Strategies on HIV, Viral Hepatitis, and STIs 2022–2030 position the health sector as central in combating epidemics but emphasize that the approach must be multisectoral, integrating health into all public policies. The goal is to overcome structural barriers and accelerate progress. Efforts should prioritize the most vulnerable populations to prevent inequalities and promote synergies between universal health coverage and primary healthcare, thereby aligning with the goals of the 2030 Agenda for Sustainable Development [21, 22]

The prevalence of these STIs varies according to region and gender. These epidemics exert a profound impact on the health and lives of children, adolescents, and adults worldwide, leading to fetal and neonatal deaths, infertility, and an increased risk of HIV infection, among other health problems [23]. Therefore, our social commitment is motivated by the dissemination of technologies to make them accessible to all social sectors, particularly to vulnerable populations. In addition to aligning with global perspectives, this study also considers the Maranhão State Health Plan, which aims to support municipalities in reducing STI incidence rates [24].

The findings of this study revealed an unfavorable socioeconomic context, with most women presenting low educational attainment, being farmers, and living with household incomes below the minimum wage. These results are consistent with the existing literature, which highlights the significant influence of a region's socioeconomic context on health behaviors and exposure to risk factors for STIs. Previous studies emphasize that low educational level, low income, and limited access to health services are key determinants in the persistence and/or emergence of infections, reinforcing risk behaviors related to STIs [25, 26].

Risky sexual behaviors, such as early sexual debut, multiple lifetime partners, and nonuse of condoms, were prevalent among the participants, reflecting patterns observed in previous studies of rural, quilombola, and Romani women. Among rural women, Mota et al. [27] reported that 62.2% did not use condoms, while Barbosa et al. [28] found that 86.2% of women also reported not using condoms, and 57.7% had an early sexual debut, with this behavior being associated with marital status. Among quilombola women, 78.7% did not use condoms [10], whereas among Romani women, this proportion reached 93.4% [16].

These behaviors are exacerbated by partners' resistance to condom use [29], early onset of sexual activity, low educational level, low income, and exposure to violence [30]. Furthermore, the low perception of vulnerability among married women and the limited use of condoms in stable relationships increase susceptibility to STIs, representing a significant challenge for public health [1, 31].

The high prevalence of STIs observed in this study (72.9%), combined with a considerable rate of coinfection (37%), is particularly concerning, especially given the predominance of *U. parvum* (61.7%) and *M. hominis* (35.3%). These agents are strongly associated with gynecological symptoms and obstetric complications, underscoring the severity of these findings [32–34]. These results are consistent with a recent study conducted in Mexico, which also identified *U. parvum* and *M. hominis* as the most prevalent agents in cervicovaginal samples, highlighting the need to include these pathogens in routine STI screening, even among asymptomatic women [23].

In contrast, the low prevalence of pathogens such as *U. urealyticum* (13.6%), *T. vaginalis* (7.1%), *C. trachomatis* (2.4%), and *M. genitalium* (0.7%), and the absence of *N. gonorrhoeae* may reflect differences in exposure and sexual behavior.

When comparing our results with previous studies, we found that the prevalence rates observed in this population are higher than those reported in earlier research, indicating significant variation in the STI prevalence among these groups. Among quilombola women from southeastern Brazil, the prevalence of at least one STI was 18.5%, with HPV (11.1%), *T. vaginalis* (6.3%), and *C. trachomatis* (4.3%) being the most frequent and no cases of *N. gonorrhoeae* [10]. Studies conducted with Romani populations reported positivity for *C. trachomatis* infection ranging from 3.8% to 7.2% [16, 35] and 13.5% for *N. gonorrhoeae* [36]. However, due to the global scarcity of studies on STIs in Romani populations, comparisons across studies remain limited.

In rural women from northeastern Brazil, the prevalence of STIs was 20%, with 11.7% for HPV, 4.5% for *C. trachomatis*, and 4.1% for *T. vaginalis*, in addition to a coinfection rate of nearly 10% [17]. These figures contrast with the 27.7% positivity for pathogens reported by Jobe et al. [37], which included 17.7% for *T. vaginalis*, 8.9% for *M. genitalium*, 8.4% for *C. trachomatis*, and 3.0% for *N. gonorrhoeae*. Conversely, Xueqiang et al. [38] reported lower prevalence rates for *Trichomonas* (2.9%) and *Chlamydia* (0.9%) and 6.6% for bacterial vaginosis, 2.6% for *U. urealyticum*, and 0.1% for *N. gonorrhoeae*, highlighting the regional variability in infection rates.

Moreover, we observed that the presence of *T.richomonas vaginalis* was statistically associated with vaginal pruritus, a relationship consistent with the existing literature. Symptomatic women with *T. vaginalis* may present with a wide range of symptoms, including genital pruritus [39]. However, it is essential to emphasize that although many vaginal symptoms were not statistically associated with etiological agents, their clinical relevance cannot be disregarded. This highlights the importance of careful, personalized clinical assessments for gynecological issues and the continuous monitoring of STIs in asymptomatic patients. Such efforts are crucial in guiding the development of more precise, effective, and accessible prevention programs, with the ultimate goal of reducing the prevalence of these infections in society [23].

Another relevant aspect identified in this study was the association between a higher number of daily baths and the presence of STIs. This finding must be interpreted with caution, considering the cultural and climatic context of the Neotropical region, where frequent hygiene practices are standard and socially encouraged. From a biological standpoint, however, excessive washing of the genital area, particularly with nonspecific soaps, may alter vaginal pH and disrupt the dominant lactobacillary microbiota, thereby creating a favorable environment for colonization by opportunistic pathogens and facilitating the acquisition of STIs [40]. Therefore, health education interventions should be culturally sensitive, promoting hygiene practices that balance cultural habits with the maintenance of vaginal health.

The factors associated with the presence of STIs in this study were partially divergent from those identified in previous research. Among quilombola women, earlier studies demonstrated that age, alcohol consumption, abnormal cytology, and bacterial vaginosis were associated with STIs [10]. In Romani women, factors such as age, sex, and ethnicity did not significantly influence the occurrence of STIs [35]. In rural women, factors such as age, lack of knowledge about STIs, extramarital or multiple sexual partners, abortions, early sexual debut, first pregnancy before the age of 16, and marital status were related to STIs. However, consistent with our findings, visiting a Pap smear clinic was also identified as an important protective factor against STIs [38, 41, 42].

This study has certain limitations, such as potential information and recall bias, given that the data provided by the women regarding their health behaviors and sexual history may have been influenced by factors such as shame or stigma associated with specific behaviors. Moreover, it is essential to consider that the study's cross-sectional design limits causal inference. Additionally, the small sample size of Romani women (*n* = 12) significantly restricts the statistical power for this specific subgroup, preventing any generalization of the results to the Romani population as a whole. The findings related to this group should therefore be interpreted as preliminary and exploratory, underscoring the urgent need for future studies with larger samples and methodological designs specifically aimed at investigating the health vulnerabilities of this population.

## 5. Conclusion

This study revealed a high prevalence of STIs among women from quilombola, rural, and Romani communities, with U. parvum and M. hominis being the most frequent agents. Overall, the women presented a low socioeconomic profile, with risky sexual practices characterized by nonuse of condoms. The analysis of risk factors identified that the absence of prior cytology, a high number of daily baths, and prolonged use of underwear were significantly associated with the presence of STIs in these populations.

The variations in STI prevalence among the different groups and the observed sociodemographic and behavioral factors highlight the complexity of sexual and reproductive health in these communities, indicating the need for differentiated and culturally sensitive approaches in public health policies. We emphasize the importance of regular screening and sexual health education. Future studies are needed to explore further the cultural and behavioral factors contributing to the high prevalence of STIs in these groups, as well as to assess the effectiveness of the implemented interventions.

The results of this study highlight the urgent need for culturally appropriate and context-specific sexual and reproductive health policies that align with the United Nations Sustainable Development Goals (SDGs). We recommend expanding access through the implementation of mobile STI screening programs that bring molecular tests (such as PCR) and counseling to rural, quilombola, and Romani communities, overcoming geographical and access barriers; culturally sensitive health education with the development of educational materials and workshops that engage with local knowledge, addressing not only condom use but also vaginal microbiome health and the potential risks of excessive intimate hygiene practices; training community health workers to act as educators and facilitators of access to health services, promoting adherence to prenatal care and preventive screening; and the systematic inclusion of vulnerable and underrepresented populations (quilombola, Romani, rural) in national epidemiological surveillance surveys of STIs, ensuring that their specific realities are considered in health planning.

## Figures and Tables

**Table 1:** Socioeconomic characteristics of quilombola, Roma, and rural women (*N* = 295).

Characteristics	*N*	Frequency (%) (95% CI)
Community type		
Quilombola	129	43.7 (38.0–49.5)
Rural	154	52.2 (46.8–58.3)
Roma	12	4.1 (2.0–6.1)
Age group (years)		
18–29	103	34.9 (29.8–40.3)
30–44	111	37.6 (31.9–43.4)
45–64	81	27.5 (22.2–32.5)
Occupation		
Housewife	23	7.8 (4.5–10.8)
Farmer	236	80.0 (74.7–84.7)
Retired	15	5.1 (2.7–7.8)
Others (student, general services, etc.)	21	7.1 (4.4–10.2)
Self-reported race/skin color		
Black	139	47.1 (41.5–52.4)
Mixed race (Parda)	149	50.5 (44.9–55.9)
White	7	2.4 (0.7–4.4)
Education level		
Illiterate	71	24.1 (19.3–28.8)
Up to lower primary education	81	27.5 (23.1–32.9)
Up to upper primary education	58	19.7 (15.6–24.1)
Incomplete secondary education	16	5.4 (3.1–8.5)
Complete secondary education	64	21.7 (16.8–26.3)
Higher education completed	5	1.7 (0.3–3.4)
Marital status		
Single	88	29.8 (24.7–34.6)
Married/stable union	196	66.4 (61.0–71.5)
Separated/divorced	4	1.4 (0.3–2.7)
Widow	7	2.4 (0.7–4.4)
Religion		
Catholic	222	75.3 (70.0–80.7)
Evangelical	52	17.6 (13.0–22.9)
No religion	16	5.4 (2.9–8.8)
Others (Adventist, Christian, Jehovah's Witness)	5	1.7 (0.3–3.1)
Family income		
No income	13	4.4 (2.0–6.8)
< 1 minimum wage	179	60.7 (54.9–66.8)
1-2 minimum wages	102	34.6 (29.0–39.5)
> 3 minimum wages	1	0.3 (0.0–1.0)

**Table 2:** Behavioral risk factors and hygiene practices among quilombola, Roma, and rural women (*N* = 295).

Risk factors	*N*	Frequency (%) (95% CI)
Alcohol use		
Yes	105	35.6 (30.2–42.7)
No	190	64.4 (57.3–69.8)
Smoking		
Yes	31	10.5 (7.3–13.9)
No	264	89.5 (86.1–92.7)
Age at sexual debut (coitarche)		
< 18 years	221	74.9 (70.0–80.0)
≥ 18 years	74	25.1 (20.0–30.0)
History of previous STI		
Yes	20	6.8 (4.1–9.7)
No	275	93.2 (90.3–95.9)
Vaginal discharge		
Yes	154	52.2 (46.8–58.6)
No	141	47.8 (41.4–53.2)
Vaginal pruritus		
Yes	61	20.7 (15.9–25.1)
No	234	79.3 (74.9–84.1)
Pelvic pain		
Yes	154	52.2 (46.1–57.1)
No	141	47.8 (42.9–53.9)
Dysuria		
Yes	41	13.9 (9.5–17.8)
No	254	86.1 (82.2–90.5)
Dyspareunia		
Yes	65	22.0 (17.6–27.0)
No	230	78.0 (73.0–82.4)
Number of lifetime sexual partners		
1	110	37.3 (31.5–42.4)
> 1	185	62.7 (57.6–68.5)
Condom use		
Always	35	11.9 (8.1–15.9)
Sometimes	68	23.1 (18.6–28.5)
Never	192	65.1 (59.5–70.2)
Contraceptive use		
Yes	52	17.6 (13.6–22.4)
No	243	82.4 (77.6–86.4)
Bathing frequency (per day)		
1–2 times	44	14.9 (10.5–19.3)
3–4 times	215	72.9 (67.5–77.6)
≥ 5 times	36	12.2 (8.6–15.8)
Intimate hygiene		
Yes	295	100.0 (100–100)
Frequency of intimate hygiene/day		
Every bath	250	84.7 (80.5–88.8)
Once a day	21	7.1 (4.7–10.0)
Twice a day	24	8.1 (5.1–11.4)
Products used for intimate hygiene		
Regular soap	156	52.9 (47.1–58.3)
Intimate or liquid soap	107	36.3 (30.8–41.9)
Coconut soap	29	9.8 (6.4–14.0)
Water only	3	1.0 (0–2.4)
Vaginal douching		
Yes	69	23.4 (19.3–28.1)
No	226	76.6 (71.9–80.7)
Underwear change		
> 1 time per day	278	94.2 (91.2–96.3)
Once a day	15	5.1 (3.1–8.1)
Not every day	2	0.7 (0–1.7)

**Table 3:** Association between symptoms and positivity for etiological agents of STIs by simple logistic regression.

Symptomatology	STI agent	*N* positive	% positive	OR (95% CI)	*p* value
Vaginal discharge	*Chlamydia trachomatis*	6	3.9	5.676 (0.675–47.740)	0.110
	*Trichomonas vaginalis*	11	7.1	1.008 (0.414–2.450)	0.987
	*Mycoplasma genitalium*	2	1.3	0.22 (0.01–4.53)^∗^	0.499^∗^
	*Mycoplasma hominis*	58	37.7	1.248 (0.772–2.016)	0.366
	*Ureaplasma parvum*	99	64.3	1.258 (0.786–2.013)	0.339
	*Ureaplasma urealyticum*	20	13.0	0.903 (0.464–1.759)	0.764

Vaginal pruritus	*Chlamydia trachomatis*	2	3.3	1.553 (0.294–8.203)	0.604
	*Trichomonas vaginalis*	8	13.1	2.566 (1.012–6.506)	**0.047**
	*Mycoplasma hominis*	26	42.6	1.486 (0.835–2.642)	0.178
	*Ureaplasma parvum*	35	57.4	0.797 (0.449–1.412)	0.437
	*Ureaplasma urealyticum*	12	19.7	1.802 (0.856–3.794)	0.121

Pelvic pain	*Chlamydia trachomatis*	3	1.9	0.680 (0.150–3.095)	0.618
	*Trichomonas vaginalis*	12	7.8	1.239 (0.506–3.037)	0.639
	*Mycoplasma genitalium*	2	1.3	0.22 (0.01–4.53)^∗^	0.499^∗^
	*Mycoplasma hominis*	51	33.1	0.822 (0.510–1.326)	0.422
	*Ureaplasma parvum*	99	64.3	1.258 (0.786–2.013)	0.339
	*Ureaplasma urealyticum*	18	11.7	0.716 (0.366–1.399)	0.328

Dysuria	*Chlamydia trachomatis*	1	2.4	1.033 (0.121–8.811)	0.976
	*Trichomonas vaginalis*	5	12.2	2.066 (0.713–5.985)	0.181
	*Mycoplasma hominis*	16	39.0	1.207 (0.612–2.380)	0.586
	*Ureaplasma parvum*	23	56.1	0.763 (0.392–1.488)	0.428
	*Ureaplasma urealyticum*	8	19.5	1.682 (0.714–3.961)	0.234

Dyspareunia	*Chlamydia trachomatis*	2	3.1	1.429 (0.271–7.539)	0.674
	*Trichomonas vaginalis*	3	4.6	0.570 (0.163–1.998)	0.380
	*Mycoplasma genitalium*	1	1.5	0.28 (0.02–4.53)^∗^	0.393^∗^
	*Mycoplasma hominis*	18	27.7	0.641 (0.350–1.175)	0.150
	*Ureaplasma parvum*	38	58.5	0.841 (0.480–1.473)	0.544
	*Ureaplasma urealyticum*	7	10.8	0.720 (0.303–1.714)	0.458

*Note:* Significant values (*p* < 0.05) are highlighted in bold.

Abbreviations: CI = confidence interval, OR = odds ratio.

^∗^Fisher's exact test was performed.

**Table 4:** Bivariate and multivariate analysis of the association between sociodemographic and behavioral factors and the presence of STIs.

Variable	Presence of STI yes *n* (%)	Presence of STI no *n* (%)	Crude OR (95% CI)	*p* value	Adjusted OR (95% CI)	*p* value
Age group				0.249		
18–29 years	80 (77.7)	23 (22.3)	1.74 (0.90–3.35)			
30–44 years	81 (73.0)	30 (27.0)	1.35 (0.72–2.52)			
45–64 years	54 (66.7)	27 (33.3)	REF			
Occupation				0.854^a^		
Farmer	172 (72.9)	64 (27.1)	REF			
Housewife	17 (73.9)	6 (26.1)	1.05 (0.40–2.79)			
Retired	12 (80.0)	3 (20.0)	1.49 (0.41–5.45)			
Others (students, general services, etc.)	14 (66.7)	7 (33.3)	0.74 (0.29–1.93)			
Education level				0.813^a^		
Illiterate	51 (71.8)	20 (28.2)	1.70 (0.26–10.95)			
Up to primary school (lower)	59 (72.8)	22 (27.2)	1.79 (0.28–11.43)			
Up to primary school (higher)	45 (77.6)	13 (22.4)	2.31 (0.35–15.32)			
Incomplete high school	10 (62.5)	6 (37.5)	1.11 (0.14–8.68)			
Complete high school	47 (73.4)	17 (26.6)	1.84 (0.28–12.00)			
Higher education	3 (60.0)	2 (40.0)	REF			
Marital status				0.531^a^		
Married/consensual union	138 (70.4)	58 (29.6)	REF			
Single	69 (78.4)	19 (21.6)	1.53 (0.84–2.76)			
Separated/divorced	3 (75.0)	1 (25.0)	1.26 (0.13–12.37)			
Widow	5 (71.4)	2 (28.6)	1.05 (0.20–5.57)			
Household income				0.264		
< 1 minimum wage	144 (75.0)	48 (25.0)	1.35 (0.80–2.30)			
> 1 minimum wage	71 (68.9)	32 (31.1)	REF			
Alcohol consumption				0.687		
Yes	78 (74.3)	27 (25.7)	1.12 (0.65–1.92)			
No	137 (72.1)	53 (27.9)	REF			
Smoking				0.548		
Yes	24 (77.4)	7 (22.6)	1.31 (0.54–3.17)			
No	191 (72.3)	73 (27.7)	REF			
Age at first sexual intercourse				0.559		
< 18 years	163 (73.8)	58 (26.2)	1.19 (0.66–2.13)			
≥ 18 years	52 (70.3)	22 (29.7)	REF			
Previous cytology^∗^				0.033		
Yes	174 (70.4)	73 (29.6)	REF		REF	
Never	41 (85.4)	7 (14.6)	2.46 (1.05–5.73)		2.39 (1.01–5.62)	0.046
Previous history of STI				0.458		
Yes	16 (80.0)	4 (20.0)	1.53 (0.49–4.72)			
No	199 (72.4)	76 (27.6)	REF			
Vaginal symptoms				0.264		
Yes	174 (74.4)	60 (25.6)	1.41 (0.77–2.60)			
No	41 (67.2)	20 (32.8)	REF			
Number of lifetime sexual partners				0.557		
1	78 (70.9)	32 (29.1)	REF			
> 1	137 (74.1)	48 (25.9)	1.17 (0.69–1.98)			
Condom use				0.564		
Always	25 (71.4)	10 (28.6)	1.00 (0.45–2.23)			
Sometimes	53 (77.9)	15 (22.1)	1.42 (0.74–2.73)			
Never	137 (71.4)	55 (28.6)	REF			
Contraceptive use				0.470		
Yes	40 (76.9)	12 (23.1)	1.30 (0.64–2.62)			
No	175 (72.0)	68 (28.0)	REF			
Daily baths^∗^				0.114		
1–2	29 (65.9)	15 (34.1)	REF		REF	
3–4	155 (72.1)	60 (27.9)	1.34 (0.67–2.67)		1.34 (0.66–2.71)	0.415
≥ 5	31 (86.1)	5 (13.9)	3.21 (1.03–9.94)		3.28 (1.04–10.33)	0.042
Intimate hygiene/day				0.641^a^		
Every bath	182 (72.8)	68 (27.2)	REF			
Once a day	14 (70.0)	6 (30.0)	0.75 (0.29–1.93)			
Twice a day	19 (79.2)	5 (20.8)	1.42 (0.51–3.95)			
Hygiene products used				0.222^a^		
Soap	114 (73.1)	42 (26.9)	REF			
Intimate soap/liquid	82 (76.6)	25 (23.4)	1.21 (0.68–2.14)			
Coconut soap	17 (58.6)	12 (41.4)	0.52 (0.23–1.18)			
Only running water	2 (66.7)	1 (33.3)	0.74 (0.07–8.34)			
Sitz baths				0.826		
Yes	51 (73.9)	18 (26.1)	1.07 (0.58–1.97)			
No	164 (72.6)	62 (27.4)	REF			
Underwear use during the day^∗^				0.026		
Yes	112 (78.9)	30 (21.1)	1.81 (1.07–3.07)		1.83 (1.07–3.12)	0.027
No	103 (67.3)	50 (32.7)	REF		REF	

^a^Fisher's exact test was applied.

^∗^Variables with *p* ≤ 0.20 were considered for the initial binary logistic regression model of the odds of “STI Positivity” vs. “STI Negativity.”

^∗∗^Model adjusted for variables significant (*p* < 0.05) in the initial model.

## Data Availability

In consideration of participant privacy, the datasets generated and/or analyzed during this study are available from the corresponding author upon reasonable request.
